# Extracts from the Records of the Boston Society for Medical Improvement

**Published:** 1858-01

**Authors:** F. E. Oliver

**Affiliations:** Secretary.


### ARTICLE XIX.

### *Extracts from the Records of the Boston Society for Medical Improvement.* By F. E. OLIVER, M. D., Secretary.

AUGUST 24th.—*Malignant Tumor of the Jaw in a Child; Death; Autopsy.* Dr. FIFIELD, of Weymouth, reported the case.

Marshall, a child, three years of age, became blind of the right eye, apparently from a cataract, within a few weeks after birth. The child was presented to the surgeons of the Boston Eye and Ear Infirmary, for operation, but the case was declined, on the ground of supposed malignancy. Shortly afterward it became blind of the left eye. The child was then shown to Dr. Henry W. Williams, who also declined operation. The general health remained good until the last week in May, 1857, when a tumor, of the size of a small chestnut, appeared on the body of the right half of the lower jaw. This was supposed to be mumps; was shown to Dr. Stevens, and by him to Drs. Lewis and Gay, by whom the diagnosis of osteo sarcoma was established. The child was then removed to West Randolph. On the 3d of July, Dr. F. saw the case for the first time. The tumor had then attained the bulk of a small orange, surrounding the jaw, distending the cheek, pushing the tongue to the left side, and rendering the mouth incapable of closure. There had been one or two attacks of hæmorrhage from the tumor.

*Sectio Cadaveris.*—A partial examination of the body was made on Sunday, August 23d. The tumor had enormously increased since the visit, filling the entire cavity of the mouth. The teeth were loosened, and easily removed by the finger. The right eye had protruded, in the form of a black fungus. On reflecting the integuments, the tumor was found to have passed the symphysis, and to have invaded the left half of the jaw, by about half the length of its body. On the right side, the body, angle, ramus and articulation had been successfully attacked and enveloped by the disease. Upward, the disease had passed under the zygoma; had penetrated the spheno-maxillary fossa. The bones comprising the base of the skull were thickly plastered with encephaloid. On attempting to remove the jaw, it was found to be spontaneously fractured at the angle. By the positive command of the parents, the removal of any portion of the tumor beyond the symphysis was forbidden.

The bulk of the whole tumor probably equalled that of a large orange, or perhaps, more correctly, a shaddock. On the removal of the fungus, representing the right eye, the walls of the orbit were seen thinly covered with encephaloid, the protrusion of the eyeball having been caused by malignant disease within itself, and not by any pressure posteriorly.

The above case is *very* remarkable in several points: first, the age at which the malignant cachexia first showed itself within the eye; secondly, the length of time it remained confined inactive, in the eye-balls; thirdly, the terrible rapidity of the growth as manifested in the jaw, it having required less than a period of three months to bring it from the size of a small chestnut to the bulk above mentioned, and to destroy life.—*Boston Med. & Sur. Jour.*

